# Increase in repulsive guidance molecule-a (RGMa) in lacunar and cortical stroke patients is related to the severity of the insult

**DOI:** 10.1038/s41598-022-24481-2

**Published:** 2022-12-01

**Authors:** Aijaz Parray, Naveed Akhtar, Ghulam Jeelani Pir, Sajitha V. Pananchikkal, Raheem Ayadathil, Fayaz Ahmad Mir, Reny Francis, Ahmed Own, Ashfaq Shuaib

**Affiliations:** 1 The Neuroscience Institute, Academic Health System, Hamad Medical Corporation, 3050 Doha, Qatar; 2 Qatar Metabolic Institute, Academic Health System, Hamad Medical Corporation, 3050 Doha, Qatar; 3 Division of Neurology, Faculty of Medicine, University of Alberta, Edmonton, T6G 2G3 Canada

**Keywords:** Cellular neuroscience, Genetics of the nervous system, Molecular neuroscience

## Abstract

Repulsive guidance molecule-a (RGMa) inhibits angiogenesis and increases inflammation. Animal models of cerebral ischemia have shown that an increased expression of RGMa leads to larger infarction and its inhibition attenuates effects of ischemia. We report on the relationship of RGMa to stroke types and severity. This is a prospective study in patients admitted to the stroke service in Qatar. We collected the clinical determinants, including NIHSS at admission, imaging and outcome at discharge and 90-days. RGMa levels were determined by measuring mRNA levels extracted from peripheral blood mononuclear cells (PBMCs) within 24 h of onset and at 5 days. There were 90 patients (lacunar: 64, cortical: 26) and 35 age-matched controls. RGMa mRNA levels were significantly higher in the stroke patients: day 1: 1.007 ± 0.13 versus 2.152 ± 0.19 [p < 0.001] and day-5: 3.939 ± 0.36 [p < 0.0001]) and significantly higher in patients with severe stroke (NIHSS ≥ 8) compared to milder symptoms (NIHSS < 8) at day 1 (NIHSS ≥ 8: 2.563 ± 0.36; NIHSS < 8: 1.947 ± 0.2) and day 5 (NIHSS ≥ 8: 5.25 ± 0.62; NIHSS < 8: 3.259 ± 0.419). Cortical stroke patients had marginally higher RGMa mRNA levels compared to lacunar stroke at day 1 (cortical stroke: 2.621 ± 0.46 vs lacunar stroke: 1.961 ± 0.19) and day 5 (cortical stroke: 4.295 ± 0.76 vs lacunar stroke: 3.774 ± 0.39). In conclusion, there is an increase in the level of RGMa mRNA in patients with acute stroke and seen in patients with lacunar and cortical stroke. The increase in RGMa mRNA levels is related to the severity of the stroke and increases over the initial 5 days. Further studies are required to determine the effects of the increase in RGMa on stroke recovery.

## Introduction

Repulsive guidance molecule-a (RGMa) is a membrane bound glycosylphosphyatidylinositol protein that was initially discovered in the visual system of the chicken embryo^1^. It is now known to be expressed in multiple organs including brain, heart, lung, liver and the small intestine^2^. RGMa binds with neogenin and, in the nervous system, has an important role in axonal guidance and survival^3^. Neuronal injury in a variety of diverse settings, including multiple sclerosis, brain trauma, stroke and spinal cord injury have been shown to have increased expression of RGMa^4^. An increased expression of RGMa may affect neuronal survival, synapse formation, growth cone collapse and immunoregulation^4^**.**

Middle cerebral artery (MCA) occlusion models of focal cerebral ischemia have shown that an increase in RGMa may increase blood brain barrier (BBB) injury and inhibition of RGMa has protective effects on the BBB^5^. In addition, blocking the increased expression of RGMa following ischemia/reperfusion in rodents has been shown to improve angiogenesis and neuronal recovery^6^. There is a recent report that an increase in RGMa was seen in acute stroke patients with MCA occlusion, and the expression was significantly higher in patients with poor collaterals^7^.

Elezanumab is a monoclonal antibody that selectively targets RGMa molecule and is in clinical trials in patients with acute stroke. In models of axonal nerve crush injury, elezanumab promotes axonal regeneration. It has also been shown to decrease inflammation in models of experimental autoimmune encephalomyelitis and improve functional recovery^8^. In the ischemic stroke patients gene expression changes in peripheral mononuclear cells (PBMCs) have been observed, suggesting these genes hold potential and could be further exploited for the diagnosis and therapeutic intervention of ischemic stroke.

We undertook the present study with the following key objectives: (a) is there an increase in the RGMa mRNA levels in peripheral blood mononuclear cells (PBMCs) in patients with acute stroke, (b) is the increase in RGMa mRNA levels evident with subcortical lacunar stroke and cortical strokes, (c) is there a relation between the expression of RGMa mRNA and the severity of the acute stroke, and finally (d) is the presence of pre-existing cerebral small vessel disease (SVD) associated with higher levels of RGMa mRNA.

## Methods

We collected clinical details on all patients with acute stroke admitted to HGH and prospectively entered it into the Qatar Stroke database. The registry was established in 2013 and now has data on more than 14,000 patients. The details of the registry have previously been published^9–11^. Any further data will be made available on request.

Patient characteristics including age, sex, nationality, medical comorbidities and prior medication were entered in the HGH Stroke registry. Data from EMS, immediate ED care, door-to-needle time (for thrombolysis patients), severity of symptoms as measured by the National Institute of Health Stroke Score (NIHSS score), length of stay (LOS) in the hospital, timing and completion of investigations, neuroimaging, post- stroke complications and in-hospital mortality was recorded for all patients. The stroke etiology was recorded according to the TOAST (Trial of Org10172 in Acute Stroke Treatment) criteria as previously described (11). Small vessel disease was measured by the methods described by Fazekas et al.^12^ and has been described in greater detail previously by our group in the population of Qatar^13^.

Patients admitted with acute stroke in the stroke ward in the Hamad General Hospital were screened based on the inclusion/exclusion criteria of the study. Following consent, blood samples at two time points were collected: initial sample within 24 h of onset of symptoms and the second one 5 days following the stroke onset. Blood samples were collected in tubes containing EDTA, and PBMCs isolated using SepMate tubes (86415, Stem Cell Technologies) and Ficoll Paque (GE 17-1440-03, GE Healthcare Life Sciences) reagent. Next, RNA was extracted from the PBMCs using All Prep DNA/RNA Mini Kit (80204, Qiagen). The RNA concentration and purity were checked using Nanodrop spectrophotometer and samples were stored at − 80 °C. Quantitative Real-Time-PCR for the expression of RGMA mRNA levels was carried out using TaqMan RNA to CT 1 Step Kit (4392938, Thermofisher) as per the manufacturer’s instructions. Briefly, 20 µl reaction was set up with 30 ng of extracted RNA samples using TaqMan Gen Expression Assays LG 30 Hs00297192 (4351368, Thermofisher) with Human GAPDH (20X) (4325792, Thermofisher) as the reference gene and run on Applied Biosystems 7500 Real-Time PCR System. The data analysis was carried out based on the comparative cycle threshold (Ct) method (2−ΔΔCt) using GAPDH as the internal standard control. Data was normalized against control and represented as fold units. (Supplementary Table 1).

Descriptive results for all quantitative variables (e.g. age) were reported as mean ± standard deviation (SD). Numbers (percentage) were reported for all qualitative variables (e.g. gender). The distribution of continuous variables was assessed prior to using statistical tools.

For statistical analyses, the software IBM SPSS Statistics ver. 26 (IBM, Armonk, USA) and GraphPad Prism 7 (GraphPad Software, Inc., LaJolla, CA, USA) were used. Samples were characterized by descriptive and inferential statistics. For normally distributed data, descriptive results (including graphical displays) are depicted as mean ± standard deviation (SD) unless mentioned otherwise. In case of non-normal distribution, results are depicted as median with interquartile range. Independent sample T-test was used to perform the bivariate analysis, and the average for all quantitative variables (e.g., age) between stroke subtypes (IS vs. SM) was compared using the Mann Whitney U-test. For multiple comparison, one-way ANOVA with Tukey’s test or t-test was used. To compare all the qualitative variables (e.g., presence or absence of CVD) between IS and SM, Pearson Chi-Square test or Fisher Exact test was used wherever appropriate. To compare differences in the cumulative incidence of MACE in 5-years among the four groups, the Kaplan–Meier survival analysis was applied, along with log rank test (Mantel-Cox Method) to test the null hypothesis that there is no difference between the four groups in the probability of an event at any time point. Hazard ratios for MACE were determined by multivariate Cox proportional hazards regression analyses to know the effect of age and other risk factors, and data presented as hazard ratio (compared with the lowest quartile) with 95% CIs.

### Patient consent and ethical approval

All patients were consented with written informed consent as per ethical approval. All the methods mentioned were in accordance with approved protocol.

The study was approved by the Institutional Review Board, Hamad Medical Corporation at the Medical Research Centre (MRC-15304/15).

## Results

There were 90 patients (Age: 47.3 ± 9.8 male: 81 females: 9) and 35 age matched (38 ± 6.5) controls available for analysis. The young age and higher number of males reflects the demographics of the State of Qatar where there is a very high population of male expatriates. The initial blood samples were taken in all stroke patients within 24 h of onset of symptoms. The stroke patients comprised of 64 subcortical lacunar stroke patients and 24 cortical stroke patients. Similar to our previous observations, there was a high incidence of diabetes, dyslipidemia and smoking in the stroke patients^9–11^. There was no significant difference in the risk factors in the lacunar or cortical stroke, except for a higher incidence of coronary artery disease in patients with cortical stroke. Patients with pre-existing small vessel disease (SVD), were older, were more likely to have diabetes and hypertension and had less severe symptoms at presentation. The lower NIHSS in patients with preexisting SVD is likely because this group had more patients with lacunar stroke at presentation. The clinical details of the stroke patients are shown in Table 1.

**Table 1:** Comparison of Stroke types (LACUNAR VS CORTICAL) with RGMA levels.

Characteristic or investigation	Total (n = 90)	Cortical Stroke Patients (n = 26, 28.9%)	Lacunar Stroke Patients (n = 64, 71.1%)	p Value
Age, Mean, years	47.3 ± 9.8	48.6 ± 10.6	46.7 ± 9.5	0.42
Sex (Male)	81 (90)	24 (92.3)	57 (89.1)	0.64
(Female)	9 (10)	2 (7.7)	7 (10.9)	
Hypertension	65 (72.2)	18 (69.2)	47(73.4)	0.69
Diabetes	40 (44.4)	11 (42.3)	29 (45.3)	0.79
Dyslipidemia	88 (97.8)	26 (100.0)	62 (96.9)	0.36
Coronary artery disease	6 (6.7)	4 (15.4)	2 (3.1)	0.03
Atrial fibrillation on admission	3 (3.3)	1 (3.8)	2 (3.1)	0.86
Active smoking	46 (51.1)	14 (53.8)	32 (50)	0.74
History of Stroke	7 (7.8)	4 (15.4)	3 (4.7)	0.09
Obesity (BMI ≥ 30 kg/m^2^)	18 (20)	2 (7.7)	16 (25)	0.06
Antiplatelet on Admission	8 (8.9)	4 (15.4)	4 (6.3)	0.38
Anticoagulant on Admission	1 (1.1)	0	1 (1.6)	0.52
Anti-Hypertensive on Admission	25 (27.8)	17 (26.6)	8 (30.8)	0.69
Anti-Diabetic on Admission	21 (23.3)	5 (19.2)	16 (25)	0.56
Statin on Admission	13 (14.4)	5 (19.2)	8 (12.5)	0.41
SVD present	69 (76.7)	20 (76.9)	49 (76.6)	0.97
NIHSS on admission	6.7 ± 3.5	7.1 ± 3.5	6.6 ± 3.6	0.51
NIHSS GROUP—< 8	59 (65.6)	16 (61.5)	43 (67.2)	0.61
≥ 8	31 (34.4)	10 (38.5)	21 (32.8)	
Length of Stay	5.7 ± 3.9	5.6 ± 1.9	5.8 ± 46	0.82
**Modified Rankin Score—at 90 days**				
0	41 (45.6)	13 (50)	28 (43.8)	0.47
1	11 (12.2)	3 (11.5)	8 (12.5)	
2	15 (16.7)	6 (23.1)	9 (14.1)	
3	17 (18.9)	2 (7.7)	15 (23.4)	
4	6 (6.7)	2 (7.7)	4 (6.3)	
5	–	–	–	
6	–	–	–	
**Prognosis—at 90-days**				
Good (mRS 0–2)	67 (74.4)	22 (84.6)	45 (70.3)	0.16
Poor (mRS 3–6)	23 (25.6)	4 (15.4)	19 (29.7)	
**NIHSS severity**				
Mild Stroke (0–4)	27 (30)	7 (26.9)	20 (31.3)	0.68
Moderate Stroke (5–10)	63 (70)	19(73.1)	44 (68.8)	
Severe Stroke (11 pr more)				

**Figure 1:**
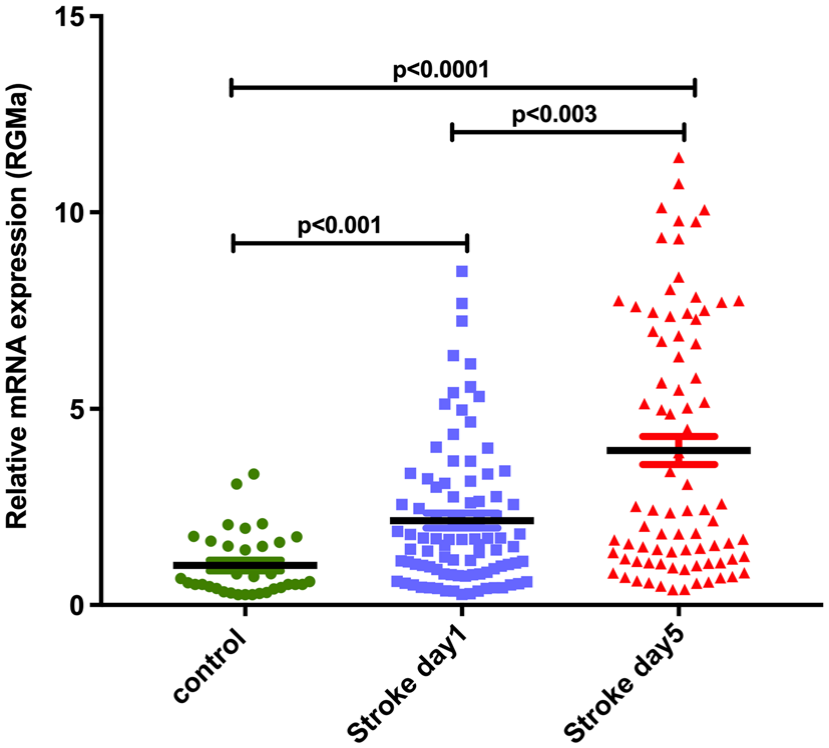
RGMA mRNA levels in control vs stroke patients on stroke onset (day1) and 5 days post stroke (day5). Data was normalized and represented as fold units (mean ± SEM). One-way ANOVA with Tukey’s test was used for multiple comparison (p < 0.05 was considered statistically significant).

**Figure 2:**
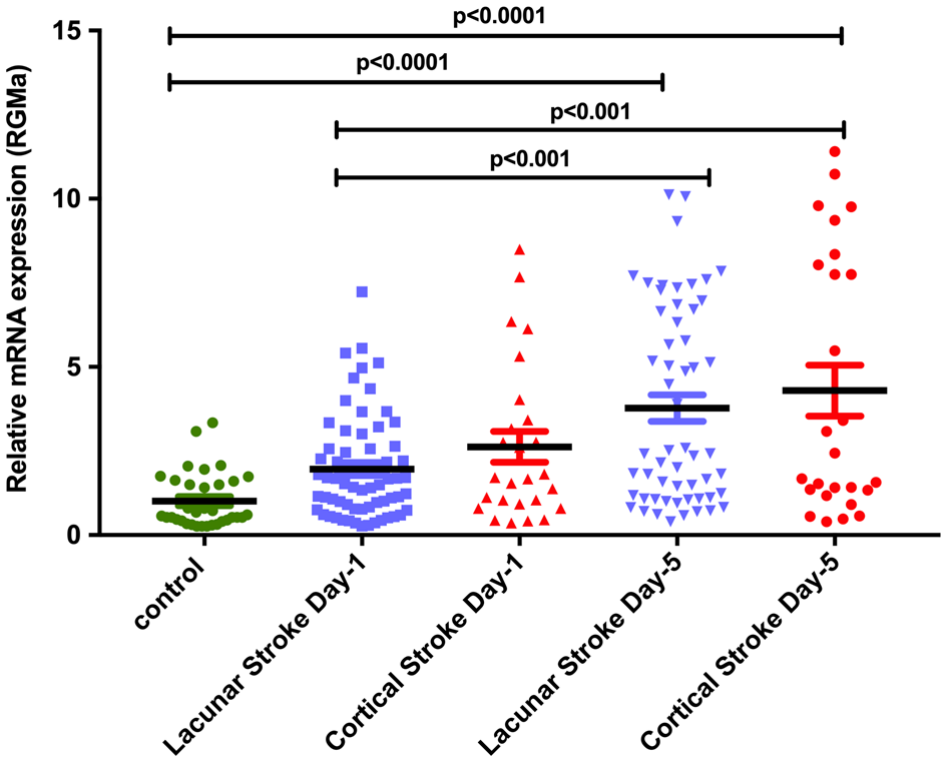
Relative RGMA mRNA levels in stroke sub-types (lacunar vs cortical) on stroke onset (day1) and 5 days post stroke (day5) in comparison to control. Data was normalized and represented as fold units (mean ± SEM). One-way ANOVA with Tukey’s test was used for multiple comparison (p < 0.05 was considered statistically significant).

Initial analysis compared RGMa mRNA levels in patients with lacunar and cortical stroke with controls at day one and day 5 following the acute stroke. There was a significant increase in the RGMa mRNA levels in both lacunar and cortical strokes on day one. The RGMa mRNA levels increased significantly on day five when compared to day one levels. The results are shown in Fig. 1. Compared to lacunar stroke, the levels of RGMa mRNA were higher in cortical stroke patients (Fig. 2).

**Figure 3:**
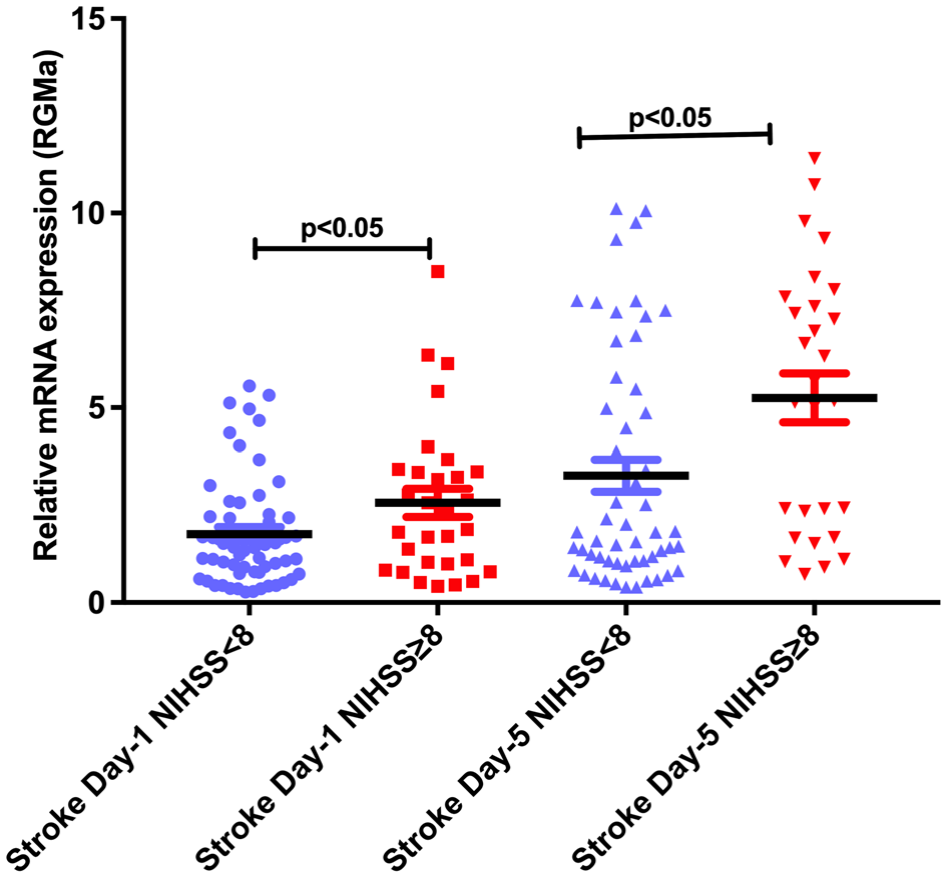
Relative RGMA mRNA levels in stroke sub-types, mild (NIHSS < 8) vs more severe stroke (NIHSS ≥ 8) on stroke onset (day1) and 5 days post stroke (day5). Data was normalized and represented as fold units (mean ± SEM). t-test was used for comparison between the stroke sub-groups (p < 0.05 was considered statistically significant).

We next compared the increase in RGMa mRNA expression in patients with milder symptoms (NIHSS < 8) to patients with more severe stroke (NIHSS ≥ 8). The NIHSS of 8 was an arbitrary cut-off for the purpose of our study. The clinical features of the two groups is shown in Table 2. There was a significant increase in the expression of RGMa mRNA in patients with NIHSS of ≥ 8 when compared to milder severity stroke as is shown in Fig. 3. We used Pearson’s correlation co-efficient test to determine the relationship between NIHSS and RGMa mRNA levels. There was a significant positive correlation between NIHSS severity and RGMa mRNA levels at day one (Pearson Correlation: 0.0239, p 0.023) and day 5 (Pearson Correlation: 0.274, p0.013). The positive correlation was seen with lacunar and cortical strokes.

**Table 2:** Stroke patients with NIHSS score severity and RGMA levels.

Characteristic or investigation	Total (n = 90)	Admission NIHSS ˂ 8 (n = 60, 66.7%)	Admission NIHSS ≥ 8 (n = 30, 33.3%)	p Value
Age, Mean, years	47.3 ± 9.8	48.4 ± 9.6	45.0 ± 9.8	0.12
Sex (Male)	81 (90)	55 (91.7)	26 (86.7)	0.46
(Female)	9 (10)	5 (8.3)	4 (13.3)	
Hypertension	65 (72.2)	45 (75)	20 (66.7)	0.40
Diabetes	40 (44.4)	31 (51.7)	9 (30.0)	0.05
Dyslipidemia	88 (97.8)	58 (96.7)	30 (100.0)	0.31
Coronary artery disease	6 (6.7)	4 (6.7)	2 (6.7)	1.00
Atrial fibrillation on admission	3 (3.3)	1 (1.7)	2 (6.7)	0.21
Active smoking	46 (51.1)	33 (55)	13 (43.3)	0.29
History of stroke	7 (7.8)	6 (10)	1 (3.3)	0.27
Obesity (BMI ≥ 30 kg/m^2^)	17 (18.9)	12 (20)	5 (16.7)	0.70
**Modified Rankin Score—at discharge**				
0	21 (23.3)	12 (20.0)	9 (30.0)	0.01
1	13 (14.4)	7 (11.7)	6 (20.0)	
2	16 (17.8)	15 (25.0)	1 (3.3)	
3	8 (8.9)	8 (13.3)	0	
4	32 (35.6)	18 (30.0)	14 (46.7)	
5	–	–	–	
6	–	–	–	
Modified Rankin Score—at 90 days				
0	52 (57.8)	33 (55.0)	19 (63.3)	0.26
1	14 (15.6)	11 (18.3)	3(10.0)	
2	11 (12.2)	8 (13.3)	3(10.0)	
3	9 (10.0)	4 (6.7)	5 (16.7)	
4	4 (4.4)	4 (6.7)	0	
5	–	–	–	
6	–	–	–	
RGMA mRNA level day 1, fold (mean ± SEM)	2.1519 ± 1.8259	1.8367 ± 1.6029	2.7519 ± 2.0872	0.02
RGMA mRNA level day 5, fold (mean ± SEM) (n = 82)	3.9391 ± 3.2382	3.2864 ± 3.0322	5.1319 ± 3.3134	0.01
**RGMA mRNA level day 1 severity**				
Mild	19 (21.1)	16 (27.1)	3 (19.7)	0.068
Moderate	37 (41.1)	25 (42.4)	12 (38.7)	
Severe	34 (37.8)	18 (30.5)	16 (51.6)	
**RGMA mRNA level day 5 severity (n = 82)**				
Mild	10 (12.2)	9 (17.0)	1 (3.4)	0.02
Moderate	29 (35.4)	22 (41.5)	7 (24.1)	
Severe	43 (52.4)	22 (41.5)	21 (72.4)

**Figure 4:**
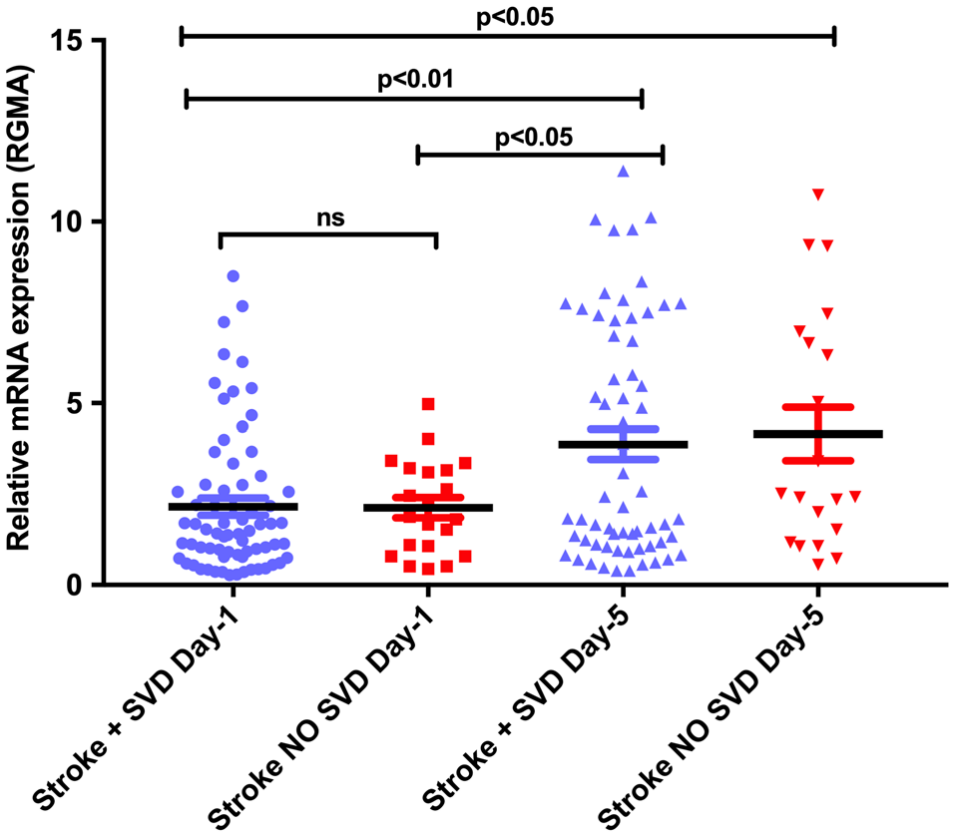
Relative RGMA mRNA levels in stroke SVD vs stroke no SVD on stroke onset (day1) and 5 days post stroke (day5). Data was normalized and represented as fold units (mean ± SEM). One-way ANOVA with Tukey’s test was used for multiple comparison (p < 0.05 was considered statistically significant; ns, not significant).

Our final objective was to determine if the presence of SVD on the MRI at the time of the stroke was associated with higher levels of RGMa mRNA. As is seen in Fig. 4, there was no significant difference in the levels of RGMa mRNA in patients with and without preexisting SVD on MRI imaging. Both groups showed similar increases in the levels of RGMa mRNA at day 1 and day 5.

## Discussion

The most important finding in our study is that the RGMa mRNA levels in Peripheral blood mononuclear cells (PBMCs) increases very early following acute stroke. This is seen both with lacunar and cortical strokes. The expression is significantly higher in cortical strokes and seems to increase over time. We were unable to show if the presence of pre-existing SVD result in higher RGMa mRNA levels. Our data also shows that SVD does not result in higher RGMa mRNA levels following acute stroke in patients with lacunar or cortical strokes on day 1 or day 5 analysis.

Neuronal recovery following ischemia, trauma and degenerative disorders require axonal outgrowth and development of new neuronal networks. RGMa is a potent inhibitor of neurite outgrowth and, through combined activation of neogenin and bone morphogenetic protein (BMP), is recognized as an important factor in inhibiting neuronal regeneration and functional recovery following central nervous system (CNS) damage^4^. In the spinal cord injury models, activated microglia inhibits axonal regrowth through a RGMa-mediated mechanism^14^. The inhibition of RGMa has been shown to improve axonal outgrowth and recovery^15^. RGMa has been studied extensively in patients with multiple sclerosis (MS). Its levels are significantly increased in patients with MS^16^ and its higher levels have been adversely related to neuronal function^17,18^.

There is considerable literature on the role of RGMa in experimental cerebral ischemia^5,6,19^. During focal cerebral ischemia, autopsy studies in humans reveal that RGMa accumulates in infarcted regions and is upregulated in neurons, reactive astrocytes and infiltrating leukocytes^19^. In a study of 25 autopsy brain specimens, RGMa-immunopositive cells accumulated in the lesion and per-lesion sites. RGMa expression was restricted to the neurons, endothelial cells and smooth muscle cells in the first week following the stroke. In more mature lesions, the RGMa expression expanded to additional cells, including activated astrocytes, fibroblastoid cells and extracellular deposition in the scar tissue^19^. RGM-immunopositive cells were also evident in the tissue surrounding intracerebral hemorrhage^19^.

There is experimental evidence that inhibition of RGMa in models of cerebral ischemia can lead to improved clinical outcome. In a rat model of focal ischemia, genetic and pharmacological suppression of RGMa resulted in significant reduction in reactive astrocytosis and glial scaring. This was associated with a pronounced improvement in neuronal function^20^. In the study of Wang et al.^6^, blocking of RGMa function by function-blocking peptide six fibronectin type (6FNIII) resulted in lower levels of RGMa in neurons and endothelial cells coupled with improved functional recovery. Minocycline inhibits RGMa expression and has been used in a study of focal ischemia in rats^21^. The decrease in RGMa levels were evident within 2 weeks of focal ischemia and was believed to be responsible for the improved BBB integrity and functional recovery^21^. Another interesting method to inhibit RGMa expression recently reported is postconditioning in ischemia–reperfusion model^22^. In addition to a reduction of the RGMa levels in the animals treated with postconditioning, there was also a significant decrease in inflammatory markers that may also contribute to the neuroprotection^22^. Finally, in a recent experiment with stimulation of the cerebellar fastigial nucleus, there was a reduction in RGMa expression and improved axonal growth following focal ischemia^23^.

To our knowledge, there is only one previous publication in patients with acute stroke where the levels of RGMa mRNA were monitored in patients following acute stroke^7^. Similar to our study, the RGMa mRNA was extracted from Peripheral blood mononuclear cells (PBMCs) within hours of arrival to the hospital in patients with cortical stroke and middle cerebral artery occlusion^7^. In their study, patients with poor cerebral pial collaterals had a higher level of RGMa mRNA. Poor pial collaterals is associated with severe stroke and a higher NIHSS^7,24^. This is similar to our results where patients with cortical stroke, especially with higher NIHSS were associated with significantly higher levels of RGMa mRNA. Together with the experimental data on the protection seen when RGMa is inhibited and the clinical data that its levels are higher in patients with severe stroke or in patients with poor pial collaterals, there is opportunity for its use in selected patients following an acute stroke.

Our study has limitations. The number of patients, especially with cortical involvement is small. The control subjects are younger and have fewer risk factors and we are unclear if this may have led to lower levels of RGMa mRNA in this group. Our data evaluated the RGMa mRNA levels in the acute time period and only up to 5 days following the stroke. It will be important to see how RGMa mRNA levels change during the weeks and months following the stroke. Such data is currently not available. Whilst we noted higher levels of RGMa mRNA in patients with previous SVD on imaging and these patients tended to have a more severe stroke, we do not have data on RGMa mRNA levels in subjects with SVD and no stroke. Such data may be useful to acquire an understanding of the mechanism of asymptomatic SVD. Similarly, we are unable to determine the effects of vascular risk factors on RGMa mRNA levels.

In summary, we have shown that there is a very early increase in the expression of RGMa mRNA in Peripheral blood mononuclear cells (PBMCs) in patients with acute stroke. Our research reveals that this is seen in lacunar and cortical stroke and is more severe in patients with higher NIHSS. There is experimental evidence for the effectiveness of inhibiting RGMa on recovery following an acute stroke. Inhibiting RGMa following an acute stroke offers a new avenue in enhancing recovery following an acute stroke.

## Supplementary Information

Supplementary Table 1.

## Data Availability

All data generated or analyzed during this study are included in this published article and its supplementary information files.

## Abbreviations

RGMa: Repulsive guidance molecule-a
PBMCs: Peripheral blood mononuclear cells
SVD: Small vessel disease
MCA: Middle cerebral artery
CVD: Cardiovascular disease
MACE: Major adverse cardiac events
